# Supplementary material for: IKKβ Inhibition Attenuates Epithelial Mesenchymal Transition of Human Stem Cell-Derived Retinal Pigment Epithelium
Source: Cells. 2023 Apr 13;12(8):1155. doi: 10.3390/cells12081155 (PMC10136838; doi:10.3390/cells12081155)
Supplement: Supplementary file 1 [file cells-12-01155-s001.zip › Supplementary Tables/cells-2121403-supplementary.pdf]

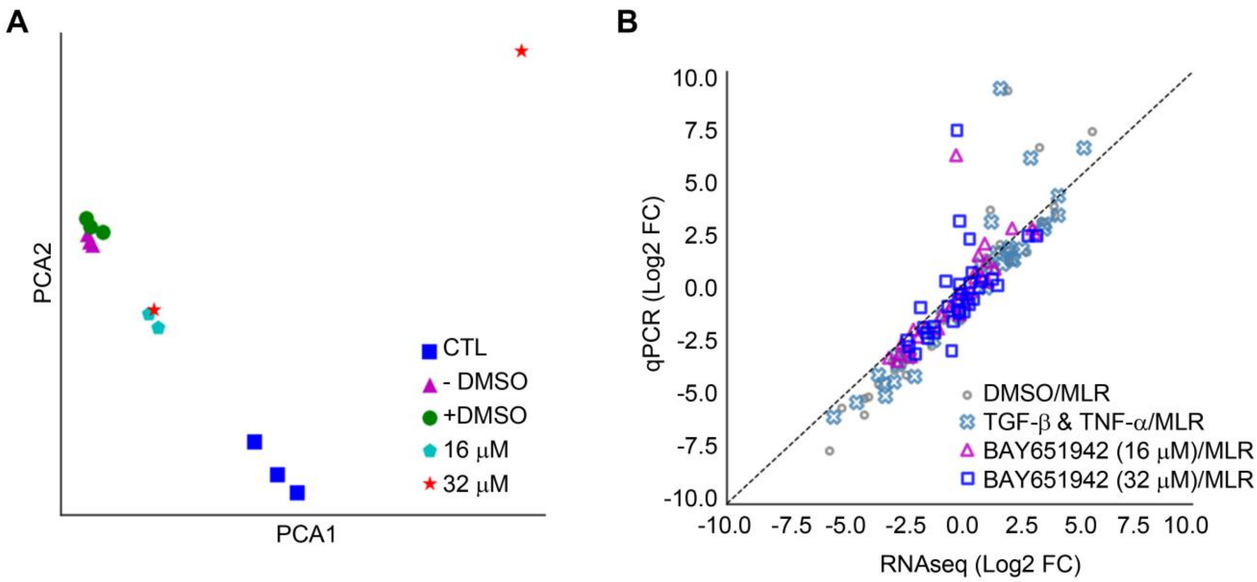

Figure S1. Correlation of RPE EMT gene expression ratios between RNA-seq and qRT PCR. (A) Principal Components Analysis (PCA) of gene expression data from dissociation and TGF-β/TNF-α treatment. (B) The expression ratios were plotted against log2 fold change value from RNA-seq (x-axis) and qRT PCR was performed using the amplified cDNA from each RNA-seq sample (y-axis). Statistically significant Pearson correlation is shown between the gene expression levels analyzed by qRT PCR and RNA-seq. (A) BAY651942/Monolayer (33 genes), (B) TGF-B/TNF-A/Monolayer (54 genes), (C) Dissociation/Monolayer (39 genes). (D-E) Principal Components Analysis (PCA) analysis of gene expression data from dissociation and TGF-β/TNF-α treatment.

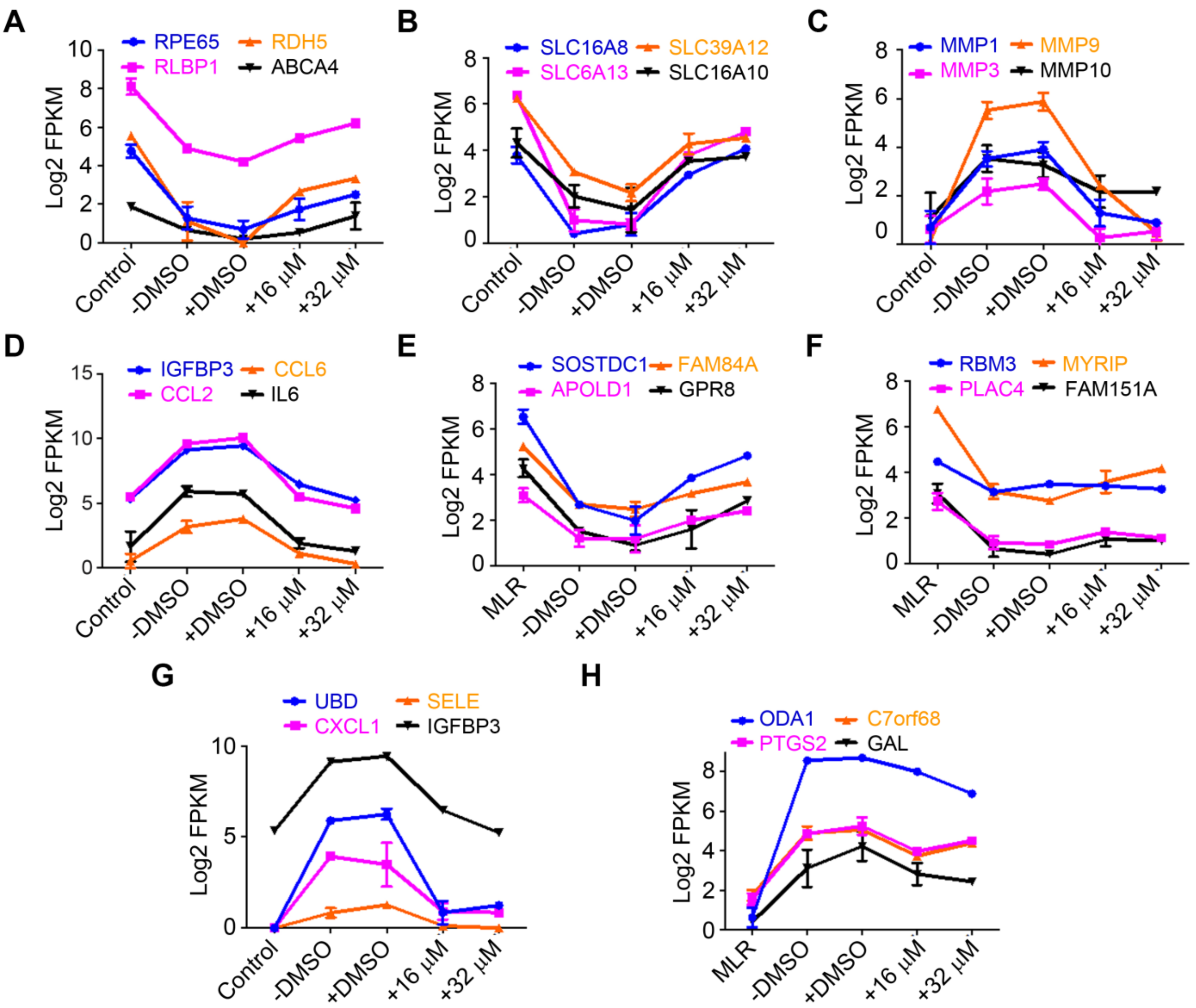

Figure S2. Differential expression of AMD associated risk factor modulated by BAY651942 treatment. A-H. The expression ratios for curated AMD associated risk genes were plotted against log2 fold change value from RNA-seq.

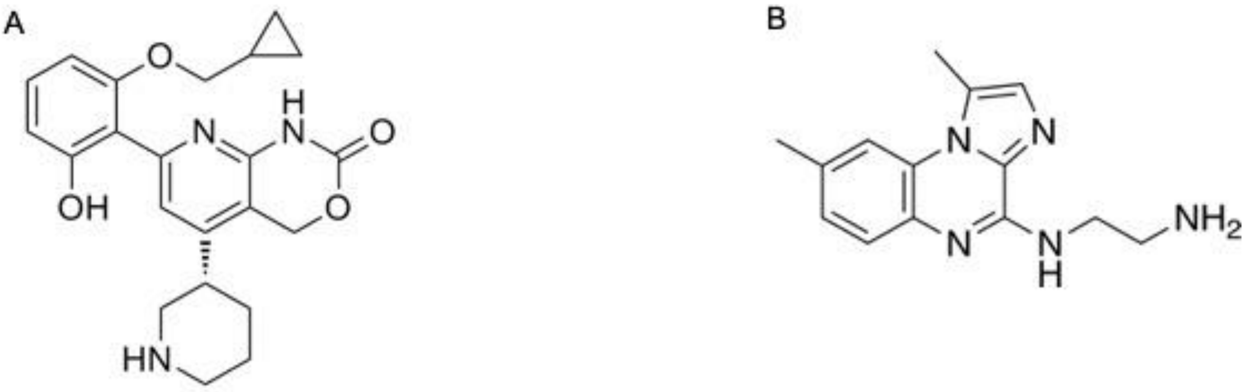

Figure S3. Chemical structure of (A) BAY 651942, and (B) BMS345541.
